# Correction to “Subclinical Ketosis: Reproductive Performance and Milk Yield in Dairy Cows Receiving Oral Glucogenic Precursors During Early Postpartum Period”

**DOI:** 10.1002/vms3.70598

**Published:** 2025-10-21

Guner, B., A. A. Erkan, B. Ozturk, et al. 2025. “Subclinical Ketosis: Reproductive Performance and Milk Yield in Dairy Cows Receiving Oral Glucogenic Precursors During Early Postpartum Period.” *Veterinary Medicine and Science* 11, no. 5: e70563. https://doi.org/10.1002/vms3.70563.

In the Materials and Methods section, the following statement was incomplete and should read as follows:

“All the procedures were approved by the **Balıkesir University** Animal Care Committee (Reference no: 2022/1‐2).”

We apologize for this error.

